# Aerosolization ocular surface microorganisms accumulation effect during non-contact tonometer measurements

**DOI:** 10.1186/s12886-024-03664-7

**Published:** 2024-09-03

**Authors:** Xinyi Shen, Yi Xu, Yuee Ye, Shuo Huai, Peiyu Wu, Jinzhi Huang, Weihe Zhou, Chunchun Li, Yanyan Chen

**Affiliations:** National Clinical Research Center for Ocular Diseases, Eye Hospital, Wenzhou Medical University, Wenzhou, 325027 China

**Keywords:** Cornea, Microbial aerosol, COVID-19

## Abstract

**Purpose:**

This study aimed to verify that aerosolization ocular surface microorganisms (AOSMs) accumulated during non-contact tonometry (NCT) measurements.

**Methods:**

A total of 508 participants (740 eyes) were enrolled in the study. In Experiment 1, before NCT was performed on each eye, the air was disinfected, and environment air control samples were collected via Air ideal^®^ 3P (Bio Merieux). During NCT measurements, microbial aerosol samples were collected once from each eye. In Experiment 2, we collected initial blank control samples and then repeated Experiment 1. Finally, in Experiment 3, after the background microbial aerosol investigation, we cumulatively sampled AOSMs from each 10 participants then culture once, without any interventions to interrupt the accumulation. The collected samples were incubated and identified using matrix-assisted laser desorption/ ionization time-of-flight mass spectrometry (MALDI-TOF-MS).

**Results:**

Pathogenic *Aerococcus viridans* and other microorganisms from human eyes can spread and accumulate in the air during NCT measurements. The species and quantity of AOSMs produced by NCT measurements can demonstrate an accumulation effect.

**Conclusion:**

AOSMs generated during NCT measurements are highly likely to spread and accumulate in the air, thereby may increase the risk of exposure to and transmission of bio-aerosols.

**Supplementary Information:**

The online version contains supplementary material available at 10.1186/s12886-024-03664-7.

## Precis

Pathogenic *Aerococcus viridans* and other species of aerosolization ocular surface microorganisms (AOSMs) can spread and accumulate with the increase of NCT measurement person times, demonstrating an accumulation effect.

**Supplementary Information:**

The online version contains supplementary material available at 10.1186/s12886-024-03664-7.

## Introduction

Microorganisms, including viruses such as severe acute respiratory syndrome(SARS), influenza and other infectious microorganisms can spread through aerosol transmission; these microorganisms often combine with aerosols to form bio-aerosols, which are then transported through the air [1–4]. Unlike respiratory droplets, bio-aerosols can remain floating in the air for extended periods of time (up to hours), allowing them to repeatedly attack the human body and increase the risk of transmission, posing a threat to human and environmental health [5].

One common method used to measure intraocular pressure (IOP), is non-contact tonometry (NCT). NCT involves the use of an air puff to flatten the cornea, resulting in the dehiscence of the tear film and the generation of aerosols [6–12]. While there are new technologies available for measuring IOP [13]. Our previous study found that the aerosols produced during NCT measurements have a cumulative effect with increasing NCT spraying times [10]. There aerosols can carry microorganisms from the ocular surface, leading to the generation of aerosolized ocular surface microorganisms (AOSMs) that are dispersed in the air [14]. The presence of AOSMs in the air could be harmful to human and environmental health.

The aim of this study was to explore the cumulative effect of AOSMs produced during NCT measurements and provide a research foundation for assessing the risk of bio-aerosol transmission during NCT measurements, as well as its impact on human and environmental health.

## Materials and methods

### Ethical approval

This single-centre observational study was approved by the Institutional Review Board of the Eye Hospital of Wenzhou Medical University (2020-018-K-16) and adhered to the tenets of the Declaration of Helsinki.

### Participants

The study comprised 508 participants (740 eyes) at The Eye Hospital, Wenzhou Medical University from December 2021 to February 2024. The inclusion criteria for participants were as follows: (a) patients requiring NCT measurement. The exclusion criteria were as follows: (a) those with a history of infectious diseases or from epidemic areas, and (b) those with contraindications for clinical NCT measurements.

### Interventions and outcomes

#### Preparation prior to the experiment

Before conducting the experiments, the experimental room was ventilated and cleaned. Additionally, we used 75% alcohol to disinfect the object surfaces and the air in the room. To minimise interference from airflow, we avoided unnecessary and intense activities and limited the frequent opening and closing of doors. The room occupied an approximate area of 30 m^2^. We set up the medical terminal (M) and patient terminal (P). The medical staff and the participants were seated at the M and P terminals, respectively.

The experiments were carried out with a consistent indoor personnel density, and only one participant was seated at the patient terminal (P). The participant remained silent and wore a mask. The experimenter wore protective gear, including masks, goggles, a white gown, and sterile hats.

We used the TX-20P Automatic NCT (Canon Co., Tokyo, Japan) to measure IOP and generate air puffs for the study of background microbial aerosols investigation. The participants were instructed to sit on the P terminal and position their chin on the mandibular support. They were instructed to keep their forehead facing forward and fixed on the forehead support. They focused on the light source in the jet port, kept their eyes open naturally, and exposed their corneas. Subsequently, both eyes of participants were measured with the NCT and repeated three times measured for each eye.

Microbial aerosols were sampled using an Air Ideal^®^ 3P (BioMérieux) sampler. The sampler allowed for an air volume of 100 L/min to pass through. The same microbial aerosol sampler was used for all the experiments. Before conducting the experiments, we sterilized the NCT and Air Ideal^®^ 3P with 75% alcohol. We used blood agar plates with a diameter of 90 mm in the sampler (Bio-kont, China).

#### Experiment 1 (Video. 1)

Before measuring the right eye of the first participant with NCT, we used 75% alcohol to disinfect the air around the NCT. Additionally, we collected 30 L of environment air control sample, labeled as 17-P-1-O1. The sampler was placed horizontally next to the air jet of the NCT. Next, we measured the right eye of the first participant with the NCT.

The sampler was positioned perpendicular to the air flow and placed horizontally next to the air jet of the NCT outside of the participant’s eye for air sampling. It was fixed on the connecting line between the air jet and the participant’s eye, leaning towards the side of the participant’s eye. The 30 L of sample collected was labeled as 17-P-1-1. After measuring each eye with the NCT, we disinfected the air jet, mandibular support, forehead support, and facial seat using cotton dipped in alcohol. We repeated the same procedure for the left eye of the first participant, and the samples were labeled as 17-P-1-O2 and 17-P-1-2, respectively. The testing order for both eyes of each participant was as follows:

1. Disinfect the environment air around the NCT, and collect an environmental air control sample before measuring the NCT of the right eye.
2. The right eye accepted NCT measured, AOSMs spread during NCT measurements sampled.
3. Disinfect the environment air around the NCT, and collect an environmental air control sample before measuring the NCT of the left eye.
4. The left eye accepted NCT measured, AOSMs spread during NCT measurements sampled.

For testing, we repeated this process 20 times to test both eyes of 20 participants. After collecting the samples, we incubated the plates in an aerobic chamber at around 25˚C for 3–5 days. Then we compared the environmental air control samples with the samples collected from the participants’ eyes and analyzed the results. The analysis of the accumulation results in Experiment 1 and Experiment 2 were based on the original data from the work of Shen et al., which we had previously published [14].

#### Experiment 2 (Video. 1)

After initially disinfecting the environment air and object surfaces, we collected initial blank air control samples.

*Initial blank air control sampling*

We collected a 30 L of air sample labeled as blank air control sample_1_ to establish the baseline level of microbial aerosols in the environment. Then, we used the NCT to produce air puffs to impact the surface of the eye model. At the same time, we sampled another 30 L of air marked as blank air control sample_2_. The sampler was placed so that it was perpendicular to the airflow at the air jet port when the initial blank control samples were collected.

*NCT measurement experimental sampling*

Before measuring the NCT of the first participant’s right eye, we disinfected the surrounding air and the NCT using 75% alcohol. We then collected a 30 L air sample labeled as 21-1-PO1. After sampling 21-1-PO1, we measured the IOP of the right eye using NCT and collected a 30 L sample during NCT measurement. The samples were marked as 21-1-P1. We repeated the same procedure for the left eye of the first participant with the samples labeled as 21-1-PO2 and 21-1-P2, respectively. The sampler was placed in the same position as in Experiment 1 during the NCT measurement.

We followed the same testing order as in Experiment 1. We repeated 41 times to test 41 participants. After sample collection, we incubated the plates at 37˚C in an aerobic chamber for 24–48 h.

#### Experiment 3 (Video. 1)

##### The background microbial aerosols investigation

*Baseline level sampling*

Before conducting the experiments, the room was cleaned, and ventilated overnight. Ultraviolet disinfection was performed, and the object surfaces and air environment were disinfected with a 75% alcohol solution at least three times to minimize the concentration of microbial aerosols in the air. Human movements were limited and kept as consistent as possible. The experimental position was kept away from the air vent. For each participant, the air jet, mandibular support, forehead support, and facial seat were disinfected using cotton dipped in alcohol. For the overall assessment of the microbial aerosols during NCT measurements, samples were taken from the M and P terminals.

We used the natural sedimentation method and opened the agar plate to sample the baseline microbial aerosols in the air when no participant was present. The plate was immediately closed, and this sample was designated as the Baseline (initial state) _1_. Then, 30 L of air was sampled during six air puffs impinging at the eye model and this sample was designated as the Baseline (air puff )_2_. The sampler was used to collect a 30 L of air sample during the first participant entered the room and sat at P terminal prior to the NCT measurements, marked as Baseline (human movements)_3_.

*Microbial aerosols sampling during air puffs*

The NCT device generated six air puffs without aiming at the participants’ eyes. They also took care to avoid touching the participants’ hair and other body parts. The sampler was positioned in the same position as in Experiments 1 and 2 during NCT measurements of participants’ eyes. Approximately 30 L of air was collected from each individual.

We cumulatively sampled microbial aerosols from 10 participants, which were then cultured once. The culture were performed after the 10th, 20th, 30th, 40th, 50th, and 60th participants from the M and P terminals, respectively. No interventions were performed to interrupt the accumulation of microbial aerosols.

After overnight air stewing, the baseline level sampling was repeated [30 L of Baseline (initial state) _1_ was collected using an Air Ideal^®^ 3P, with other procedures same as above]. Then we cumulatively sampled microbial aerosols from each 10 participants without NCT measurements, which were then cultured once. The culture were performed from the M and P terminal, respectively, this part was same as above.

##### AOSMs sampling during NCT measurements

Baseline level sampling repeated. Then NCT produced air pulses lead to the dehiscence of the tear film during IOP measurements for both eyes of each participant. Approximately 30 L of air was collected from each individual using the sampler.

We cumulatively sampled from 10 participants, which were then cultured once. The culture were performed after the 10th, 20th, 30th, 40th participants and so on from the M and P terminal, respectively. No interventions were performed to interrupt the accumulation during the experiment.

A total of 310 participants (618 eyes) were enrolled to repeat the accumulation experiment. Plates in rounds 4 and round 5 were kept in aerobic conditions at about 35 °C for 24–48 h. Blood agar plates in other rounds were kept in aerobic conditions at approximately 25 °C for 3–5 days after sample collection.

#### Identification

The species of colonies were identified using the matrix-assisted laser desorption/ ionization time-of-flight mass spectrometry (MALDI TOF MS). We performed cluster analysis for Rounds 3–5 using the Autof Analyzer software included in the MALDI-TOF MS (Autof MS 600, Autobio, China). A double-blind study was conducted, with the experimental sampling personnel distinct from those responsible for identification.

#### Statistical analysis

Microsoft Office 4.3.4.14 (Microsoft, Redmond, Wash, USA) and Epidata 3.1 (The EpiData Association, Denmark) was used to establish a database for parallel double input. Statistical analyses were performed by IBM SPSS Statistics (version 25.0; IBM Corp., Armonk, NY, USA).

## Results

In Experiment 1, the following microorganisms were found to spread and accumulate in the air after NCT measurements were performed on 20 participants (40 eyes): *Psychrobacter faecalis*,* Kocuria palustris*,* Staphylococcus epidermidis*,* Chryseobacterium*,* Stenotrophomonas acidaminiphila*,* Brevundimonas*,* Pseudomonas otitidis*,* Chryseobacterium arachidiradicis*,* Chryseobacterium indologenes* (Video 2).

In Experiment 2, the following microorgansims were found to accumulate after NCT measurements were performed on 41 participants (82 eyes): *Kocuria palustris*,* Bacillus megaterium*,* Bacillus subterraneus*,* Aerococcus viridans*,* Enterobacter hormaechei*,* Sporosarcina* (Video 2).

The number of microbial aerosols quantitatively accumulated and increased with an increase in the number of participants NCT measured in Experiments 1–2 (see Supplementary Table S1).

In Experiment 3, after cluster analysis, the species of AOSMs may accumulate with an increase in the number of NCT measurements performed (Video 2). Compared to the baseline (background), the number of microbial aerosols quantitatively accumulated and increased with an increase in the number of participants NCT measured (see Supplementary Table S1).

## Discussion

The results suggested that AOSMs generated during NCT measurements are highly likely to spread and accumulate in the air. This may be due to the cumulative effect of aerosols produced during NCT measurements [10]. Supporting this, our previous study demonstrated that microorganisms from the ocular surface can attach to the aerosols produced during NCT measurements, leading to the generation and dispersion of AOSMs in the air [14, 15]. As the number of NCT measurements increased, the number of microbial aerosols also accumulated, indicating that these microbial aerosols may persist in the air and pose a risk to human health over time. In light of these findings, it is essential to implement measures to mitigate pollution risks.

Although basic disinfection measures, such as ultraviolet light and alcohol, were found to have some effectiveness in reducing AOSMs in final samples, we recommend enhancing these practices. Specifically, using ultraviolet rays for 30 min and 75% alcohol to disinfect the environment and object surfaces at least three times may help decrease the basic of aerosols and bio-aerosols in the air to a certain level; and during the following aerosol generation procedure (AGP), the bio-aerosols level in the air can’t increase quickly and highly. These recommendations align with the findings of Shang et al., who identified ultravioletray for 30 min as the optimal inactivation time and timely inactivation is therefore recommended [16].

However, during NCT measurements, there is still an accumulation of AOSMs in the air. This accumulation poses an increased risk of microbial aerosols to human health. Therefore, it is urgent to propose a novel method to continuously and stably decrease the bio-aerosols produced during NCT measurements. This will help maintain a stable and safe level of bio-aerosols in the air. One possible solution is to use layer-by-layer self-assembly technology to synthesize polymer nanocomposites that can inactivate microorganisms and reduce the risk of microbial aerosols to protect human health [17]. Further more, we can formulate dopamine in situ reduction of nano silver and other anti-microorganism solution by making device. Poly(acrylic acid)(PAA)(4 g, 35 wt%), 1-ethyl-3-(3-dimethylaminopropyl) carbodiimide (EDC)(0.2 g), dopamine hydrochloride (0.35 g), and N-Hydroxysuccinimide(NHS) (0.05 g) were dissolved in 50 mL of deionized water; stirring then continued for 8 h; the product was obtained after dialyzing for 72 h and freeze-drying at -20 °C [17]. Poly (ethyleneimine) (branched polyethylenimine [PEI] combined with silver nitrate. Finally, PAA-dopa (0.4 mg/mL, pH = 3.2) and PEI-Ag+ (0.4 mg/mL, pH = 10.8, AgNO_3_ 0.05 mg/mL) can be prepared as two solutions and their buffer solutions with the same pH value [17]. They can be layer-by-layer modified the baffles on NCT, other medical equipment and any other AGP conditions to decrease microbial aerosols. Notably, the formulation of dopamine for in situ reduction of nano silver is portable and can be used worldwide. And other Nano Silver formula also have excellent or even better effect of anti-microbial aerosols and aerosols [18].

When NCT measurements involve more than 20 participants, it is recommended to disinfect at least tree times using anti-microorgansims solutions after every ten participants, or to disinfect more frequently, in order to inactivate and prevent airborne microorganisms and eliminate cumulative effects during AGP. Moreover, while Next-Generation Sequencing (NGS) metagenomics is a new technology that can achieve extremely accurate and efficient detection of microorganisms, it was not necessary for our experiment [19].

The limitation of our study is that in the future, more new and harmless materials should be developed to reduce the accumulation of aerosols and microbial aerosols in various fields. Additionally, future studies should utilize models such as Quantitative Microbial Risk Assessment (QMRA) to further estimate the risk of aerosol-borne illnesses caused by pathogenic microorganisms such as SARS as well as to evaluate the impact of risk mitigation efforts, in order to provide references for decreasing the risk of pathogenic microorganism aerosol transmission [20–22].

## Conclusion

There is a high likelihood that AOSMs produced during NCT measurements can accumulate with an increase in the number of NCT measurement person times, thereby may increase the risk of microbial aerosol transmission to human health.

## Electronic supplementary material

Below is the link to the electronic supplementary material.

**Supplementary Material 1: Video 1**: The video of the experiments procedure in the study

**Supplementary Material 2: Video 2**: The video of the experiments results

**Supplementary Material 3: Supplementary Table S1**: The numbers and properties of microbial aerosols produced during NCT measurements. Supplementary Table S1 sequentially displayed the number and properties of microbial aerosols produced in Experiment 1-2; the number of microbial aerosols produced in Experiment 3 background and Rounds 1-5; the number of microbial aerosols produced during non-contact tonometer measurements in our prior experiments

## Data Availability

No datasets were generated or analysed during the current study.

## References

[1] Liu Z, Liu H, Zhang M, He J, Cao G. Experimental study on the generation of aerosol particles and microorganisms from surgical staff in an operating room. Build Environ. 2023;236:110266. 10.1016/j.buildenv.2023.110266

[2] Leung NHL. Transmissibility and transmission of respiratory viruses. Nat Rev Microbiol. 2021;19(8):528–45. 10.1038/s41579-021-00535-6. Epub 2021 Mar 22. PMID: 33753932; PMCID: PMC7982882. 33753932 10.1038/s41579-021-00535-6 PMC7982882

[3] Chen PZ, Bobrovitz N, Premji Z, Koopmans M, Fisman DN, Gu FX. Heterogeneity in transmissibility and shedding SARS-CoV-2 via droplets and aerosols. Elife. 2021;10:e65774. 10.7554/eLife.65774. PMID: 33861198; PMCID: PMC8139838. 33861198 10.7554/eLife.65774 PMC8139838

[4] Fennelly KP. Particle sizes of infectious aerosols: implications for infection control. Lancet Respir Med. 2020;8(9):914–24. 10.1016/S2213-2600(20)30323-4. Epub 2020 Jul 24. PMID: 32717211; PMCID: PMC7380927. 32717211 10.1016/S2213-2600(20)30323-4 PMC7380927

[5] Zuo YY, Uspal WE, Wei T. Airborne transmission of COVID-19: aerosol dispersion, lung deposition, and virus-receptor interactions. ACS Nano. 2020;14(12):16502–24. 10.1021/acsnano.0c08484. Epub 2020 Nov 25. PMID: 33236896. 33236896 10.1021/acsnano.0c08484

[6] Britt JM, Clifton BC, Barnebey HS, Mills RP. Microaerosol formation in noncontact ‘air-puff’ tonometry. Arch Ophthalmol. 1991;109(2):225-8. 10.1001/archopht.1991.01080020071046. PMID: 1993032. 10.1001/archopht.1991.01080020071046 1993032

[7] Yousefi A, Ma Y, Roberts CJ, Moroi SE, Reilly MA. Hydrodynamic interaction between tear film and air puff from noncontact tonometry. Transl Vis Sci Technol. 2022;11(2):2. PMID: 35103798; PMCID: PMC8819281. 35103798 10.1167/tvst.11.2.2 PMC8819281

[8] Chen X, Li B, Zhang C. Assessment of temporal and spatial distribution patterns of aerosol produced by air-puff non-contact tonometer. Eur J Ophthalmol. 2022;32(5):3012–3018. doi: 10.1177/11206721211054727. Epub 2021 Dec 3. PMID: 34859700. 10.1177/11206721211054727 34859700

[9] Guo H, Li W, Huang Y, Li X, Li Z, Zhou H, Sun E, Li L, Li J. Increased microbial loading in aerosols produced by non-contact air-puff tonometer and relative suggestions for the prevention of coronavirus disease 2019 (COVID-19). PLoS ONE. 2020;15(10):e0240421. 10.1371/journal.pone.0240421. PMID: 33031477; PMCID: PMC7544126. 33031477 10.1371/journal.pone.0240421 PMC7544126

[10] Li C, Tang Y, Chen Z, Wang A, Huang X, Chen Y, et al. Aerosol formation during non-contact air-puff tonometry and its significance for prevention of COVID-19. Chin J Exp Ophthalmol. 2020;38(3):212–6.

[11] Shetty R, Balakrishnan N, Shroff S, Shetty N, Kabi P, Roy D, Joseph SM, Khamar P, Basu S, Sinha Roy A. Quantitative high-speed assessment of droplet and aerosol from an eye after impact with an air-puff amid COVID-19 scenario. J Glaucoma. 2020;29(11):1006–1016. 10.1097/IJG.0000000000001672. PMID: 32947358. 10.1097/IJG.0000000000001672 32947358

[12] Tang Y, Li C, Chen Y, Chen Z, Zhang P, Wang A, Huang X, Qu J, Li M, Ma S, Vasudevan B. Effect of intraocular pressure on aerosol density generated by noncontact tonometer measurement. J Glaucoma. 2020;29(11):1001–1005. 10.1097/IJG.0000000000001669. PMID: 32941321. 10.1097/IJG.0000000000001669 32941321

[13] Borroni D, Gadhvi KA, Hristova R, McLean K, Rocha de Lossada C, Romano V, Kaye S. Influence of corneal visualization Scheimpflug technology tonometry on intraocular pressure. Ophthalmol Sci. 2021;1(1):100003. 10.1016/j.xops.2021.100003. PMID: 36246003; PMCID: PMC9562332. 36246003 10.1016/j.xops.2021.100003 PMC9562332

[14] Shen X, Xu Y, Huang J, Wu P, Zhou W, Chen Y. A comparative study on two methods of ocular surface microbial sampling. BMC Ophthalmol. 2023;23(1):228. 10.1186/s12886-023-02979-1. PMID: 37217905; PMCID: PMC10201025. 37217905 10.1186/s12886-023-02979-1 PMC10201025

[15] Shen X. 2024. URL: https://twitter.com/sxy19980826/status/1790655248253800843?s=19. Accessed 15 May 2024.

[16] Shang M, Kong Y, Yang Z, Cheng R, Zheng X, Liu Y, Chen T. Removal of virus aerosols by the combination of filtration and UV-C irradiation. Front Environ Sci Eng. 2023;17(3):27. 10.1007/s11783-023-1627-y. Epub 2022 Sep 7. PMID: 36118139; PMCID: PMC9470504. 36118139 10.1007/s11783-023-1627-y PMC9470504

[17] Wang B, Ye Z, Xu Q, et al. In situ construction of Ag NPs in bio-inspired multilayer films for long-term bactericidal and biofilm inhibition properties. Polym Test. 2017;62(2017):162–70. 10.1016/j.polymertesting.2017.06.023 10.1016/j.polymertesting.2017.06.023

[18] Ju Y, Han T, Yin J, Li Q, Chen Z, Wei Z, Zhang Y, Dong L. Bumpy structured nanofibrous membrane as a highly efficient air filter with antibacterial and antiviral property. Sci Total Environ. 2021;777:145768. 10.1016/j.scitotenv.2021.145768. Epub 2021 Feb 15. PMID: 33684755; PMCID: PMC7954306. 33684755 10.1016/j.scitotenv.2021.145768 PMC7954306

[19] Parekh M, Borroni D, Romano V, Kaye SB, Camposampiero D, Ponzin D, Ferrari S. Next-generation sequencing for the detection of microorganisms present in human donor corneal preservation medium. BMJ Open Ophthalmol. 2019;4(1):e000246. 10.1136/bmjophth-2018-000246. PMID: 31179394; PMCID: PMC6528759. 31179394 10.1136/bmjophth-2018-000246 PMC6528759

[20] Clements N, Arvelo I, Arnold P, Heredia NJ, Hodges UW, Deresinski S, Cook PW, Hamilton KA. Informing building strategies to reduce infectious aerosol transmission risk by integrating dna aerosol tracers with quantitative microbial risk assessment. Environ Sci Technol. 2023;57(14):5771–81. 10.1021/acs.est.2c08131. Epub 2023 Mar 31. PMID: 37000413. 37000413 10.1021/acs.est.2c08131

[21] Schijven J, Vermeulen LC, Swart A, Meijer A, Duizer E, de Roda Husman AM. Quantitative microbial risk assessment for airborne transmission of SARS-CoV-2 via breathing, speaking, singing, coughing, and sneezing. environ health perspect. 2021;129(4):47002. 10.1289/EHP7886. Epub 2021 Apr 1. Erratum in: Environ Health Perspect. 2021;129(9):99001. 10.1289/EHP10105. PMID: 33793301; PMCID: PMC8016178. 10.1289/EHP7886 PMC8016178 33793301

[22] Henriques A, Mounet N, Aleixo L, Elson P, Devine J, Azzopardi G, Andreini M, Rognlien M, Tarocco N, Tang J. Modelling airborne transmission of SARS-CoV-2 using CARA: risk assessment for enclosed spaces. Interface Focus. 2022;12(2):20210076. 10.1098/rsfs.2021.0076. PMID: 35261732; PMCID: PMC8831086. 35261732 10.1098/rsfs.2021.0076 PMC8831086

